# Synthesis of New Quinoxalines Containing an Oxirane Ring by the TDAE Strategy and *in Vitro* Evaluation in Neuroblastoma Cell Lines

**DOI:** 10.3390/molecules190914987

**Published:** 2014-09-18

**Authors:** Marc Montana, Florian Correard, Omar Khoumeri, Marie-Anne Esteve, Thierry Terme, Patrice Vanelle

**Affiliations:** 1 Aix-Marseille Université, CNRS, Institut de Chimie Radicalaire ICR, UMR 7273, Laboratoire de Pharmaco-Chimie Radicalaire, Marseille 13385, France; E-Mails: marc.montana@univ-amu.fr (M.M.); omar.khoumeri@univ-amu.fr (O.K.); thierry.terme@univ-amu.fr (T.T.); 2 Aix-Marseille Université, INSERM, CRO2, UMR_S911, Marseille 13385, France; E-Mails: florian.correard@gmail.com (F.C.); marie-anne.esteve@univ-amu.fr (M.-A.E.); 3 AP-HM, Hôpital Timone, Pharmacie, Marseille 13005, France

**Keywords:** quinoxaline, TDAE, XK469, isatin, oxirane, neuroblastoma, anti-cancer drugs

## Abstract

Neuroblastoma is an aggressive pediatric malignancy with significant chemotherapeutic resistance. In order to obtain new compounds active on neuroblastoma cell lines, we investigated the reactivity of carbanion formed via TDAE in quinoxaline series. The new synthesized compounds were tested for their anti-proliferative activity on two neuroblastoma cell lines, and seven oxirane derivatives obtained interesting activities.

## 1. Introduction 

Neuroblastoma is a neuroendocrine tumor that remains a major therapeutic challenge in pediatric oncology. Treating it requires aggressive multimodal therapy. As response rates to chemotherapy are low, surgery remains the only effective treatment. However, since many tumors have metastasized at the time of diagnosis, long-term survival rates for children with advanced disease are poor, and undesirable side effects frequently occur. Accordingly, there is a substantial need for new therapeutic options.

**Scheme 1:**
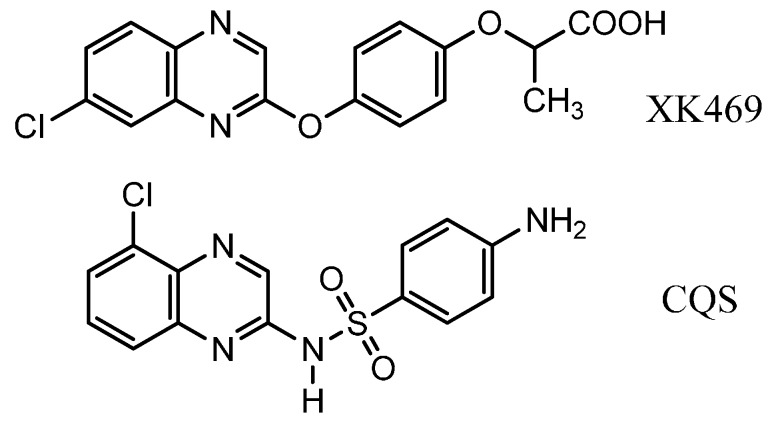
Structures of XK469 and chloroquinoxaline sulfonamide (CQS).

The quinoxaline derivatives show very interesting biological properties (anti-viral [1], anti-cancer [2], anti-leishmanial [3]) and are far from having revealed their full potential in medicinal chemistry [4]. Many drug candidates bearing quinoxaline core structures are in clinical trials in anti-viral [1], anti-cancer [2], anti-bacterial and CNS (central nervous system) therapeutic areas. Among them, XK469 and chloroquinoxaline sulfonamide (CQS) are known as anti-neoplastic quinoxaline topoisomerase II inhibitors [5,6,7] (see Scheme 1). XK469, or (±)-2-[4-(7-chloro-2-quinoxaliny)oxy] phenoxy propionic acid, is an analog of the herbicide, Assure^®^, synthesized by DuPont Company, which possesses anti-tumor activity.

The quinoxaline anti-tumor agent, XK469, inhibited neuroblastoma tumor growth in a 14-year-old with relapsed neuroblastoma, who experienced disease stabilization for 14 months [8].

Tetrakis(dimethylamino)ethylene (TDAE) is a reducing agent, which reacts with halogenated derivatives to generate, under mild conditions, an anion via two sequential transfers of one electron [9,10,11,12]. We have shown that, from *o*- or *p*-nitrobenzyl chloride, TDAE can generate a nitrobenzyl carbanion, which is able to react with various electrophiles, such as aromatic aldehydes [13], ketones, α-keto-esters, α-ketolactams and diethyl ketomalonate [14,15,16].

In continuation of our research program centered on the design and synthesis of novel bioactive molecules [17,18,19,20,21], we decided to explore the anti-proliferative potential of quinoxaline compounds.

## 2. Results and Discussion

### 2.1. Chemistry

In the present study, 13 quinoxalines were evaluated against human neuroblastoma (SK-N-SH and IMR-32—SK-N-SH and IMR-32 are not acronyms but the appellations of cell lines) cell lines, 12 of which were prepared in our lab (clinical compound XK469 was purchased). Apart from molecules **8**, **15**, **16**, all synthesized molecules bear an oxirane moiety. Compounds synthesized after molecules **3b** and **4a** were observed to have an interesting activity against SK-N-SH and IMR-32 cell lines. Thus, a structural homogeneous quinoxaline series was obtained, presenting a structural analogy with XK469. 

**Scheme 2:**
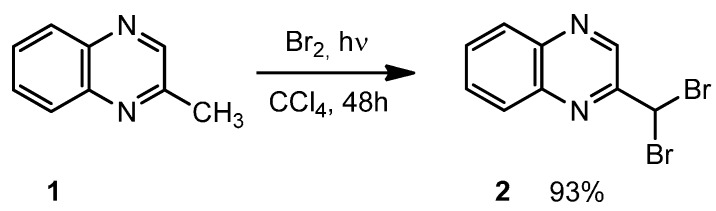
Synthesis of 2-(dibromomethyl)quinoxaline **2**.

The 2-(dibromomethyl)quinoxaline **2** was synthesized in one step from 2-methylquinoxaline **1**, as previously described (Scheme 2) [22].

**Scheme 3:**
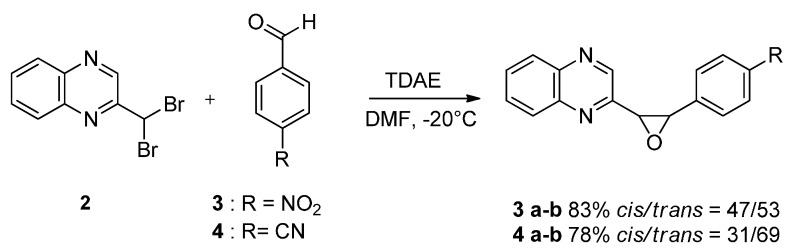
Reactivity of aromatic aldehydes in the presence of TDAE.

Oxiranes **3**–**4a**–**b** resulted from the reaction of 2-(dibromomethyl)quinoxaline with aromatic aldehydes in the presence of TDAE (Scheme 3) [22].

**Scheme 4:**
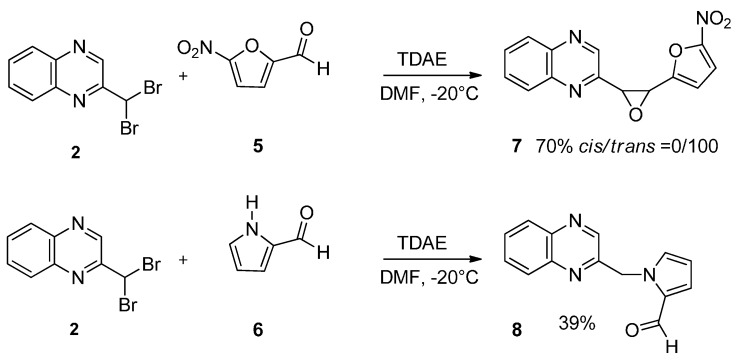
Reactivity of heterocyclic aldehydes in the presence of TDAE.

Our next step was to test whether the nature of the aldehyde changed the biological effect on neuroblastoma cell lines. Thus, we investigated the reaction of 2-(dibromomethyl)quinoxaline **2** with three equivalents of aldehydes **5**–**6** in the presence of TDAE at −20 °C for 1 h, followed by 2 h at room temperature. Only *trans*-oxirane **7** and unexpected 1-(quinoxalin-2-ylmethyl)-1*H*-pyrrole-2-carbaldehyde **8** were isolated (Scheme 4). 

**Scheme 5:**
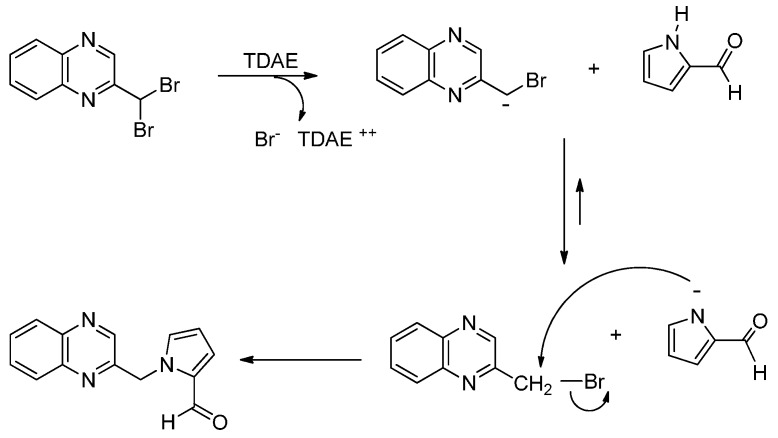
Reactivity of 1*H*-pyrrole-2-carbaldehyde in the presence of TDAE.

The α-bromo carbanion formed by the action of the TDAE could react as a base with the 1*H*-pyrrole-2-carbaldehyde, leading to the corresponding pyrrolic anion and to the 2-bromomethyl-quinoxaline. The formation of Compound **8** could thus result from a nucleophilic substitution between these two species (Scheme 5).

**Scheme 6:**
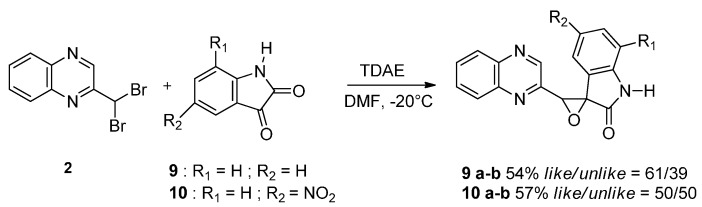
Reactivity of various isatin derivatives in the presence of TDAE.

To study the effect of steric hindrance, we investigated the reaction of 2-(dibromomethyl)quinoxaline **2** with two isatin derivatives, **9**–**10**, in the presence of TDAE under classical TDAE conditions. These reactions led to a mixture of like/unlike-isomers of corresponding oxiranes **9**–**10a**–**b** in good yields, as shown in Scheme 6. 

**Scheme 7:**
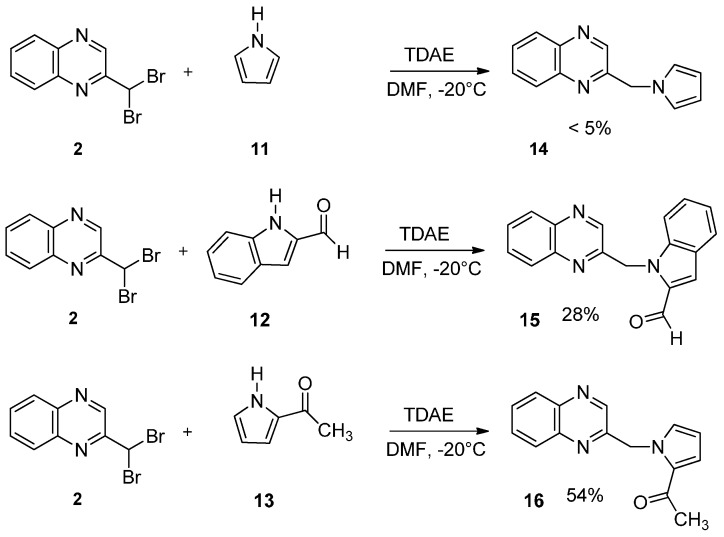
Reactivity of various carbonyl pyrrole in the presence of TDAE.

Diastereoisomers were separable, and their configuration was identified by NMR-analysis from the γ-left effect, as previously described [23]. Finally, the impact of the oxirane group on biological activity was studied. Thus, the reaction observed with Compound **6** was extended to other pyrrole derivatives (Scheme 7).

### 2.2. In Vitro Anti-Proliferative Activity of Quinoxalines Derivatives against Cancer Cell Lines

Quinoxalines **3a**–**b**, **4a**–**b**, **7**, **8**, **9a**–**b**, **10a**–**b**, **15**, **16** and clinical compound XK469 were evaluated for their *in vitro* anti-proliferative activity on a panel of SK-N-SH and IMR-32 cancer cell lines, using the conventional microculture tetrazolium reduction assay [24,25,26].

Results of these evaluations are summarized in Table 1.

**Table 1:** Anti-proliferative activity of quinoxaline derivatives.

N°	Structure	SK-N-SH Cell Line	IMR-32 Cell Line
		IC_50_ ± SD (μM)	IC_50_ ± SD (μM)
XK469	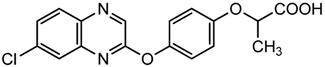	4.6 ± 1.0	13.0 ± 2.9
**3a** *cis*-isomer	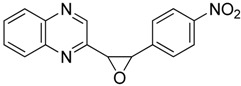	93.0 ± 10.5	64.5 ± 9.7
**3b** *trans*-isomer	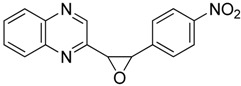	16.0 ± 1.2	14.7 ± 1.4
**4a** *cis*-isomer	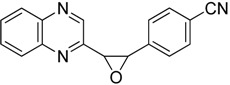	32.5 ± 1.9	25.9 ± 2.4
**4b** *trans*-isomer	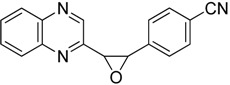	> 100	45.9 ± 7.2
**7** *trans*-isomer	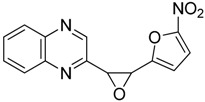	3.9 ± 0.2	5.0 ± 0.9
**8**	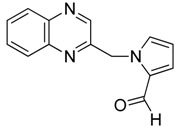	>100	>100
**9a** *like*-isomer	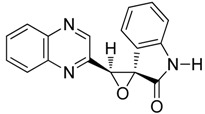	25.0 ± 3.1	15.4 ± 4.8
**9b** *unlike*-isomer	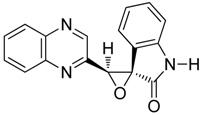	27.2 ± 6.4	14.0 ± 2.8
**10a** *like*-isomer	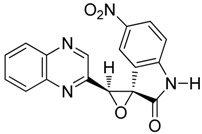	21.8 ± 1.5	7.3 ±1.6
**10b** *unlike*-isomer	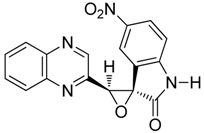	18.9 ± 4.1	9.7 ± 2.7
**15**	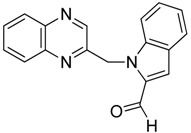	>100	>100
**16**	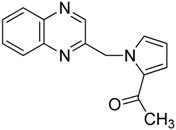	>100	>100

The IC_50_ (concentration that inhibits 50% of cell proliferation) values show clearly that the presence of an oxirane nucleus is necessary to obtain anti-proliferative activity on the two tested neuroblastoma cell lines. The reaction between the 2-(dibromomethyl)quinoxaline **2** and the pyrrole derivatives led to three compounds, **8**, **15**, **16**, for which no activity was observed on either of the cell lines at the maximal tested dose. All oxirane derivatives displayed substantial anti-proliferative activity towards neuroblastoma cell lines. These results confirm that the oxirane group played a key role in the anti-proliferative activity [27,28,29,30]. 

Compound **7** demonstrated a better activity than XK469, used as the reference drug, on SK-N-SH cells (IC_50_ = 3.9 ± 0.2 μM *vs.* 4.6 ± 1.0 μM, respectively) and IMR-32 cells (IC_50_ = 5.0 ± 0.9 μM *vs.* 13.0 ± 2.9 μM, respectively).

This first study on only four couples of stereoisomers does not allow us to end on the importance of the stereochemistry on the activity. The synthesis and the evaluation of new couples of stereoisomers with an oxirane group can allow one to appreciate the importance of the stereochemistry.

This initial structure activity relationship (SAR) study will be pursued. The nature of the heterocyclic aldehyde and the use of azaheterocycles other than quinoxaline are currently under investigation. 

## 3. Experimental Section 

### 3.1. General

Melting points were determined on a Büchi B-540 and are uncorrected. Elemental analyses were carried out on an Interscience Flash EA 1112 series (Thermo Finnigan, San Jose, CA, USA) elemental analyzer at the Spectropole, Faculté des Sciences, site de Saint-Jérôme. Both ^1^H- and ^13^C-NMR spectra were determined on a Bruker Avance 200 spectrometer (operating at 200 MHz for ^1^H and 50 MHz for ^13^C). ^1^H and ^13^C-NMR shifts (δ) were reported in parts per million (ppm) with respect to CDCl_3_ 7.26 ppm for ^1^H and 77.0 ppm for ^13^C and DMSO-*d*_6_ 2.50 for ^1^H and 39.7 ppm for ^13^C. Multiplicities were represented by s (singlet), d (doublet), t (triplet), q (quartet) and m (multiplet). The adsorbent used for column chromatography was: silica gel 60 (Merck, Darmstadt, Germany, 230–400 mesh). Thin-layer chromatography was performed with Merck 60F-254 silica gel (0.25 mm layer thickness) in an appropriate solvent.

The general procedure for the reaction of 2-(dibromomethyl)-quinoxaline **2** with various aldehydes **3**–**6** or isatin and pyrrolic derivatives **9**–**13**, using TDAE: 

To a two-necked flask equipped with a silica-gel drying tube and a nitrogen inlet was added, under nitrogen at –20 °C, 10 mL of anhydrous DMF solution of 2-(dibromomethyl)-quinoxaline 2 (0.45 g, 1.5 mmol) and various aldehydes **3**–**6** or isatin and pyrrolic derivatives **9**–**13**, (4.5 mmol, 3 equivalents). The solution was stirred and maintained at this temperature for 30 min, and then the TDAE (0.3 g, 1.5 mmol) was added dropwise (via a syringe). A red color immediately developed with the formation of a white fine precipitate. The solution was vigorously stirred at −20 °C for 1 h and then warmed up to room temperature for 2 h. After this time, TLC analysis (dichloromethane) clearly showed that Compound 2 was totally consumed. The orange-red turbid solution was filtered and hydrolyzed with 80 mL of H_2_O. The aqueous solution was extracted with chloroform (3 × 40 mL), and the combined organic layers were washed with H_2_O (3 × 40 mL) and dried over MgSO_4_. Evaporation of the solvent left an orange solid as a crude product. Purification by silica gel chromatography (5/5 dichloromethane/ethyl acetate) and recrystallization from ethanol gave the corresponding derivatives. 

*4-(3-quinolanin-2-yl-oxiranyl)benzonitrile* **4a**, pink solid, m.p. 161.5 °C; ^1^H-NMR (CDCl_3_): δ 4.24 (d, *J* = 4.3 Hz, 1H, CH); 4.33 (d, *J* = 4.3 Hz, 1H, CH); 7.52 (d, *J* = 8.6 Hz, 2H, 2×CH); 7.72 (d, *J* = 8.6 Hz, 2H, 2×CH); 7.84 (m, 2H, 2×CH); 8.14 (m, 2H, 2×CH); 8.88 (s, 1H, CH); ^13^C-NMR (CDCl_3_): δ 59.2 (2×CH), 112.2 (C), 118.2 (C); 127.4 (2×CH); 128.8 (CH), 129.4 (CH); 130.2 (CH); 130.6 (CH); 132.0 (2×CH); 138.5 (C); 141.5 (C); 142.0 (CH); 142.8 (C); 151.0 (C). Elemental analysis calculated: C, 74.71; H, 4.06; N, 15.38; elemental analysis found: C, 74.47; H, 4.22; N, 15.05.

*4-(3-quinolanin-2-yl-oxiranyl)benzonitrile* **4b**, pink solid, m.p. 178.5 °C ^1^H-NMR (CDCl_3_): δ 4.61 (d, *J* = 1.9 Hz, 1H, CH); 4.71 (d, *J* = 1.9 Hz, 1H, CH); 7.48 (m, 2H, 2×CH); 7.74 (m, 4H, 4×CH); 8.01 (m, 2H, 2×CH); 8.66 (s, 1H, CH); ^13^C-NMR (CDCl_3_): δ 60.8 (CH); 62.1 (CH); 112.7 (C); 118.4 (C); 126.4 (2×CH); 129.2 (CH); 129.5 (CH); 130.4 (CH); 130.8 (CH); 132.6 (2×CH); 141.3 (C); 141.9 (C); 142.1 (CH); 142.7 (C); 150.3 (C). Elemental analysis calculated: C, 74.71; H, 4.06; N, 15.38; elemental analysis found: C, 74.29; H, 4.23; N, 15.07.

*2*-[3-(5-Nitrofuran-2-yl)oxiran-2-yl]*quinoxaline* **7**, maroon solid, m.p. 171 °C ^1^H-NMR (CDCl_3_): δ 4.44 (d, *J* = 1.9 Hz, 1H, CH); 4.74 (d, *J* = 1.9 Hz, 1H, CH); 6.77 (d, *J* = 3.7 Hz, 1H, CH); 7.34 (d, *J* = 3.7 Hz, 1H, CH); 7.81 (m, 2H, 2×CH); 8.12 (m, 2H, 2×CH); 8.89 (s, 1H, CH); ^13^C-NMR (CDCl_3_): δ 54.0 (CH); 59.4 (CH); 112.2 (CH); 112.5 (CH); 129.2 (CH); 129.5 (CH); 130.6 (CH); 130.9 (CH); 141.9 (CH); 142.4 (C); 142.8 (C);149.1 (C); 152.5 (C). Elemental analysis calculated: C, 59.37; H, 3.20; N, 14.84; elemental analysis found: C, 59.75; H, 3.23; N, 14.52.

*1-(quinoxalin-2-ylmethyl)-1H-pyrrole-2-carbaldehyde* **8**, black solid, m.p. 275 °C ^1^H-NMR (DMSO): δ 5.88 (s, 2H, CH_2_); 6.32 (m, 1H, CH); 7.01 (m, 1H, CH); 7.18 (s, 1H, CH); 7.72 (m, 2H, 2×CH); 8.04 (m, 2H, 2×CH); 8.63 (s, 1H, CH); 9.55 (s, 1H, CH); ^13^C-NMR (DMSO): δ 54.1 (CH_2_); 110.7 (CH); 125.1 (CH); 129.1 (CH); 129.2 (CH); 130.0 (C); 130.2 (CH); 131.4 (C); 132.0 (CH); 141.7 (C); 142.0 (C); 144.0 (CH); 151.8 (C); 179.7 (CH=O). Elemental analysis calculated: C, 70.87; H, 4.67; N, 17.71; elemental analysis found: C, 70.90; H, 4.81; N, 17.71.

*3'-(quinoxalin-2-yl)spiro[indoline-3,2'-oxiran]-2-on* **9a**, orange powder, m.p. 197 °C ^1^H-NMR (DMSO): δ 5.32 (s, 1H); 6.94 (m, 1H); 7.10 (m, 1H); 7.50 (m, 3H) 7.84 (m, 2H); 8.00 (m, 1H); 9.15 (s, 1H); 10.82 (s, 1H); ^13^C-NMR (DMSO): δ 62.21 (C); 64.53 (CH); 112.67 (CH); 122.62 (C); 123.24 (CH); 125.17 (CH); 129.04 (CH); 129.50 (CH); 130.88 (CH); 130.98 (CH); 138.85 (CH); 141.09 (C); 141.73 (C); 143.80 (C); 144.72 (CH); 151.19 (C); 171.07 (C=O). Elemental analysis calculated: C, 70.58; H, 3.83; N, 14.52; elemental analysis found: C, 70.58; H, 4.01; N, 14.24.

*3'-(quinoxalin-2-yl)spiro[indoline-3,2'-oxiran]-2-one* **9b**, orange powder, m.p. 212 °C ^1^H-NMR (DMSO): δ 5.00 (s, 1H); 6.64 (m, 2H); 6.92 (m, 1H); 7.21 (m, 1H); 7.93 (m, 2H); 8.14 (m, 2H); 9.19 (s, 1H); 11.00 (s, 1H); ^13^C-NMR (DMSO): δ 62.65 (C); 63.75 (CH); 111.38 (CH); 120.53 (C); 122.25 (CH); 123.98 (CH); 129.39 (CH); 129.63 (CH); 131.25 (CH) 131.28 (CH); 131.55 (CH); 141.28 (C); 142.02 (C); 144.52 (C); 145.19 (CH); 149.32 (C); 171.91 (C=O). Elemental analysis calculated: C, 70.58; H, 3.83; N, 14.52; elemental analysis found: C, 69.94; H, 3.99; N, 14.12.

*5-nitro-3'-(quinoxalin-2-yl)spiro[indoline-3,2'-oxiran]-2-one* **10a**, beige powder, m.p. 250 °C ^1^H-NMR (DMSO): δ 5.63 (s, 1H); 7.13 (m, 1H); 7.15 (m, 2H); 8.01 (m, 1H); 8.11 (m, 1H); 8.35 (m, 2H); 9.16 (s, 1H); 11.50 (s, 1H); ^13^C-NMR (DMSO): δ 61.61 (C); 64.66 (CH); 111.52 (CH); 119.63 (CH); 123.87 (C); 128.19 (C); 129.11 (CH); 129.52 (CH); 131.10 (CH); 141.13 (C); 141.82 (C); 143.17 (C); 144.64 (CH); 148.75 (CH); 149.92 (CH); 171.53 (C=O). Elemental analysis calculated: C, 61.08; H, 3.02; N, 16.76; elemental analysis found: C, 61.68; H, 3.06; N, 16.76.

*5-nitro-3'-(quinoxalin-2-yl)spiro[indoline-3,2'-oxiran]-2-one* **10b**, beige powder, m.p. 230 °C ^1^H-NMR (DMSO): δ 5.13 (s, 1H); 7.08 (m, 1H); 7.95 (m, 2H); 8.20 (m, 4H); 9.22 (s, 1H); 11.63 (s, 1H); ^13^C-NMR (DMSO): δ 61.92 (C); 64.12 (CH); 111.21 (CH); 120.88 (CH); 121.95 (C); 127.82 (C); 129.21 (CH); 129.67 (CH); 131.37 (CH); 140.99 (C); 142.20 (C); 142.47 (C); 146.77 (CH); 148.98 (CH); 150.79 (CH); 156.47 (C-NO_2_); 172.58 (C=O). Elemental analysis calculated: C, 61.08; H, 3.02; N, 16.76; elemental analysis found: C, 61.08; H, 3.54; N, 16.54.

*1-(quinoxalin-2-ylmethyl)-1H-indole-3-carbaldehyde* **15**, black powder, m.p. 116 °C ^1^H-NMR (DMSO): δ 5.95 (s, 2H); 7.24 (s, 2H); 7.60 (m, 1H); 7.82 (m, 2H); 7.94 (m, 2H); 8.10 (m, 1H); 8.54 (s, 1H); 8.97 (s, 1H); 9.97 (s, 1H); ^13^C-NMR (DMSO): δ 50.45 (CH_2_); 111.71 (CH); 118.20 (C); 121.61 (CH); 123.15 (CH); 124.26 (CH); 125.24 (C); 129.31 (CH) 129.43 (CH); 130.79 (CH); 131.19 (CH); 137.77 (C); 141.52 (C); 141.81 (C); 142.15 (CH); 145.25 (CH); 152.21 (C); 185.41 (C=O). Elemental analysis calculated: C, 75.25; H, 4.56; N, 14.63; elemental analysis found: C, 74.98; H, 4.68; N, 14.62.

*1-(1-(quinoxalin-2-ylmethyl)-1H-pyrrol-2-yl)ethanone* **16**, black powder, m.p. 72 °C ^1^H-NMR (DMSO): δ 2.28 (s, 3H); 5.83 (s, 2H); 7.20 (m, 1H); 7.41 (m, 1H); 7.81 (m, 2H); 7.95 (m, 1H); 8.04 (m, 1H); 8.50 (s, 1H); ^13^C-NMR (DMSO): δ 27.27 (CH_3_); 52.79 (CH2); 109.11 (CH); 121.43 (CH); 129.13 (CH); 129.35 (CH); 130.24 (CH); 130.27 (C); 130.91 (CH) ; 132.79 (CH); 141.45 (C); 141.48 (C); 144.57 (CH); 154.12 (C); 188.22 (C=O). Elemental analysis calculated: C, 71.70; H, 5.21; N, 16.72; elemental analysis found: C, 71.14; H, 5.17; N, 16.58.

### 3.2. In Vitro Biological Evaluation

Drugs: Stock solutions of quinoxaline were prepared in dimethylsulfoxide (Sigma). Stock solutions were aliquoted and stored at −20 °C. For culture and experiments in living cells, drugs were freshly diluted at appropriate concentrations in culture medium. 

Cell culture: Neuroblastoma cancer cells, namely SK-N-SH and IMR-32 cells, were routinely maintained in standard RPMI 1640 (l-glutamine +) culture medium (Invitrogen) supplemented with 10% fetal bovine serum (Lonza) and 1% penicillin and streptomycin (Invitrogen). All cells were routinely maintained at 37 °C and 5% CO_2_.

Growth experiments: Exponentially growing cells (37,500 cells/cm²) were seeded in 96-well plates for 24 h for SK-N-SH cells and for 72 h for IMR-32 cells and then incubated with the drugs for 72 h. The number of viable cells was estimated using the colorimetric 3-(4,5-dimethylthiazol-2-yl)-2,5-diphenyltetrazolium bromide (MTT; Sigma) assay according to our previous work [17]. After drug treatment, the medium was replaced by fresh medium containing MTT (0.5 mg/mL), and cells were incubated at 37 °C for 2 h. Then, the MTT solution was removed, and DMSO was used to dilute the formazan crystals formed in the surviving cells. Finally, absorbance was measured at 600 nm with a Multiskan (Ascent) plate reader. Inhibiting concentrations were graphically determined. IC_50_ is defined as the concentration that inhibits 50% of cell proliferation. At least three independent experiments (in triplicate) were performed, and data were expressed as the mean ± SD.

## 4. Conclusion

Having previously demonstrated how the TDAE strategy can be used to obtain a series of highly functionalized quinoxalinic oxiranes, we present herein an original reactivity initiated by TDAE with compounds possessing labile hydrogen. Biological evaluation of these synthesized compounds revealed promising anti-proliferative activity toward SK-N-SH and IMR-32 cell lines for most of the compounds bearing an oxirane group. Biological results showed that Compound **7** is more active than XK-469, a quinoxaline derivative involved in clinical trials, confirming that the oxirane group plays a key role in the anti-proliferative activity. 

Further investigation on SAR and the mechanism of the anti-proliferative activity of these compounds will be carried out in next works. Moreover, due to promising results observed with Compound **7**, it will be tested as potential anti-tumor drug on other cell lines.
